# Opposite effects of HIF-1α and HIF-2α on the regulation of IL-8 expression in endothelial cells

**DOI:** 10.1016/j.freeradbiomed.2011.08.023

**Published:** 2011-11-15

**Authors:** Urszula Florczyk, Szymon Czauderna, Anna Stachurska, Magdalena Tertil, Witold Nowak, Magdalena Kozakowska, Lorenz Poellinger, Alicja Jozkowicz, Agnieszka Loboda, Jozef Dulak

**Affiliations:** a Department of Medical Biotechnology, Faculty of Biochemistry, Biophysics, and Biotechnology, Jagiellonian University, 30–387 Krakow, Poland; b Department of Cell and Molecular Biology, Karolinska Institutet, Stockholm, Sweden

**Keywords:** AdHIF-1α/AdHIF-2α, adenoviral vectors containing HIF-1α or HIF-2α cDNA, respectively, ARE, antioxidant-response element, ARNT, aryl hydrocarbon receptor nuclear translocator, GFP, green fluorescent protein, HIF, hypoxia-inducible factor, HO-1, heme oxygenase-1, IL-8, interleukin-8, NQO1, NAD(P)H:quinone oxidoreductase, SEAP, secreted alkaline phosphatase, siRNA, small interfering RNA, TP, thymidine phosphorylase, VEGF, vascular endothelial growth factor, Angiogenesis, SP-1, c-Myc, Transcription factor, Free radicals

## Abstract

Recently we have shown that hypoxia as well as overexpression of the stable form of hypoxia-inducible factor-1α (HIF-1α) diminished the expression of interleukin-8 (IL-8) by inhibition of the Nrf2 transcription factor in HMEC-1 cells. Because HIF isoforms may exert different effects, we aimed to examine the influence of HIF-2α on IL-8 expression in endothelial cells. In contrast to HIF-1α, overexpression of HIF-2α obtained by adenoviral transduction resulted in increased expression of IL-8 in an Nrf2-independent way. Importantly, HIF-2α augmented the activity of SP-1, a transcription factor involved in IL-8 regulation and known coactivator of c-Myc. Additionally, HIF-1 decreased, whereas HIF-2 increased, c-Myc expression, and silencing of Mxi-1, a c-Myc antagonist, restored IL-8 expression downregulated by HIF-1α or hypoxia. Accordingly, binding of c-Myc to the IL-8 promoter was abolished in hypoxia. Importantly, both severe (0.5% O_2_) and mild (5% O_2_) hypoxia diminished IL-8 expression despite the stabilization of both HIF-1 and HIF-2. This study reveals the opposite roles of HIF-1α and HIF-2α in the regulation of IL-8 expression in endothelial cells. However, despite stabilization of both isoforms in hypoxia the effect of HIF-1 is predominant, and downregulation of IL-8 expression in hypoxia is caused by attenuation of Nrf2 and c-Myc.

## Graphical abstract

**Figure:**
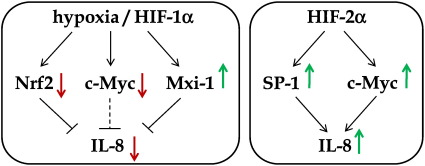

**Highlights:**

► HIF-1 decreases whereas HIF-2 increases the expression of IL-8 in endothelial cells. ► SP-1 and c-Myc are involved in the HIF-2α-dependent IL-8 upregulation. ► Mxi-1, a c-Myc antagonist, mediates IL-8 diminishment by hypoxia/HIF-1α. ► Inhibition of Nrf2 activity by hypoxia/HIF-1α adds to the downregulation of IL-8. ► Both HIF isoforms are stabilized in hypoxia but the effect of HIF-1α is predominant.

Hypoxic transcriptional response is primarily mediated by the hypoxia-inducible factors (HIF-1 and -2), both consisting of α and β subunits. In contrast to the β subunit, also known as the aryl hydrocarbon receptor nuclear translocator (ARNT), which is constitutively expressed, the α subunit is degraded at normal oxygen tension [1]. HIF-1α and HIF-2α are characterized by similar structures. Both are members of the family of basic helix–loop–helix and PER–ARNT–SIM domain-containing transcription factors [2,3]. Apart from these domains, important for DNA binding and dimerization with HIF-1β, respectively, a central oxygen-dependent degradation domain and two transactivation domains, the N-terminal activation domain (NAD) and C-terminal activation domain (CAD), are recognized in their structures [4]. NAD confers target gene specificities of HIF-1α and HIF-2α, whereas the CAD promotes the expression of their common target genes [5].

Under hypoxia, the formation of an active HIF complex from α and β subunits occurs in concert with certain factors. The p300/CREB-binding protein is a central integrating coactivator [6], which, in a hypoxia-dependent manner, owing to its binding the CAD of HIF-1α or HIF-2α, enables the recruitment of various accessory cofactors, such as the steroid receptor coactivator, transcription intermediary factor-2, or redox factor Ref-1 [4,6]. Interestingly, specific coactivators have been found, which take part exclusively in the formation of transcriptional complexes regulating target genes of HIF-2α such as the NF-κB essential modulator enhancing HIF-2α activity at normoxia [7], Ets1 regulating the transcription of vascular endothelial growth factor (VEGF) receptor 2 [8], and Elk-1 regulating erythropoietin (EPO), CITED-2, and the plasminogen activator inhibitor-1 [9].

Both HIF α subunits recognize the same DNA sequence (5′-(A/G)CGTG-3′), termed the hypoxia response element (HRE), found within the promoters or enhancers of target genes. However, differences in the NAD of the HIF isoforms and the requirement of specific transcriptional cofactors imply they may regulate the expression of distinct proteins, despite their high structural similarities. Thus, expression profiling and functional studies have revealed that HIF-1α and HIF-2α regulate both shared and unique target genes. The most common shared target is VEGF. On the other side, HIF-1α exclusively stimulates the expression of several glycolytic enzymes, whereas the embryonic transcription factors Oct-4, cyclin D1, and EPO are upregulated under hypoxia in a HIF-2α-dependent manner [10–15]. Regulation of gene expression by HIF α subunits currently seems to be more complex, because of the revealed interaction of HIF-1α/HIF-2α and c-Myc/Max proteins. HIF-2α augments c-Myc activity by stabilizing the c-Myc:Max complex, which promotes cell cycle progression. In contrast, HIF-1α inhibits the c-Myc function, resulting in cell cycle arrest at the G1/S phase [16]. Such action of HIF-1α may be explained by its binding to the Max protein and competition with c-Myc, inhibition of c-Myc protein stability [17], alteration of interaction with the SP-1 transcription factor known as the coactivator of c-Myc [16], or induction of the c-Myc antagonist, Mxi-1 [18]. Importantly, Mxi-1 has been found to be induced by hypoxia, in a HIF-1α-dependent manner, with concomitant downregulation of c-Myc target genes [19,20].

It is well known that hypoxia is a potent inducer of the formation of new blood vessels by enhancing the expression of VEGF. Interestingly, we have recently shown that the expression of interleukin-8 (IL-8), another mediator of angiogenesis, tumor growth, and metastasis, is downregulated by HIF-1α via attenuation of the Nrf2 transcription factor expression and activity [21]. Moreover, IL-8 has been found to be significantly increased in Mxi-1 knockdown cells [20]. As HIF-1α and HIF-2α may differ in their functions and as HIF-1α antagonizes the c-Myc action [18], by enhancing Mxi-1 expression, we hypothesized that the effects of both subunits on IL-8 expression may be opposite.

In this study we report that in contrast to HIF-1α, overexpression of HIF-2α resulted in increased expression of IL-8. Accordingly, HIF-2α enhanced the activity of SP-1, which may regulate IL-8. Furthermore, we revealed that c-Myc is involved in the observed effect of HIF-2α on IL-8 upregulation, whereas Mxi-1 partially mediated the opposite effect of HIF-1α. The different functions of the HIF α isoforms in the regulation of IL-8 may add to our understanding of inflammatory processes, hypoxic response, and further blood vessel formation.

## Materials and methods

### Cell culture and incubation experiments

Human microvascular endothelial cells (HMEC-1) were cultured under standard conditions (5% CO_2_, 37 °C, 95% humidity) as described previously [21]. Briefly, they were incubated in MCDB 131 medium (Gibco) containing 10% fetal bovine serum, L-glutamine (2 mM), epidermal growth factor (10 ng/ml), hydrocortisone (1 μg/ml), and antibiotics, 50 U/ml penicillin and 50 μg/ml streptomycin.

Hypoxia chambers were used as previously described [21]. In brief, after a change for fresh medium, culture dishes were placed in a humidified airtight incubator with inflow and outflow valves and a hypoxic gas mixture (0.5% O_2_, 5% CO_2_, and balance N_2_). Where indicated, 5% O_2_ was used in the hypoxic gas mixture. Then the chamber was kept at 37 °C for 6 or 24 h. At the same time, control normoxic cells were placed in an incubator containing 21% O_2_, 5% CO_2_, 74% N_2_, at 37 °C.

### Plasmid transfection

Plasmid transfections were performed in cells growing to 60–80% confluence in 24-well plates, using 0.5 μg of DNA mixed with 2.5 μl of SuperFect (Qiagen) per well, according to the vendor's protocol. After 2.5 h the cells were washed and overlaid with a regular culture medium for 24 h and, where indicated, were transduced with adenoviral vectors for the next 48 h or incubated under hypoxia for the next 24 h.

The plasmid containing the full-length promoter of the IL-8 gene driving luciferase expression was a kind gift from Dr. Rainer de Martin (Vienna, Austria). The construct containing the full-length promoter of the VEGF gene driving luciferase expression was kindly provided by Dr. Hideo Kimura (Chiba, Japan) [22]. The pNFκB-SEAP and pAP-1-SEAP vectors, containing the NF-κB and AP-1 binding regions connected to the secreted alkaline phosphatase (SEAP) reporter gene, were purchased from Clontech. The construct containing the antioxidant response element (ARE) sequences driving the expression of luciferase was kindly supplied by Dr. J.A. Johnson (University of Wisconsin, Madison, WI, USA) [23]. The SP-1-luc plasmid, containing the upstream region of the VEGF promoter from − 133 to + 3 bp cloned into a pAH1409 vector, was kindly supplied by Dr. Ulrike Fiedler (Tumor Cell Biology, Freiburg, Germany). The pCMV-lacZ vector containing the β-galactosidase gene driven by the CMV promoter was purchased from Promega and was cotransfected into cells together with one of the above-described reporter plasmids as an internal control. The enzyme activity of luciferase was determined in cell lysates according to the manufacturer's instructions (Promega). The enzyme activity of SEAP was assessed in culture medium using a chemiluminescence assay according to the vendor's protocol (Clontech). The activity of SEAP or luciferase was normalized to β-galactosidase activity. Additionally, a pBluescript vector encoding SP-1 transcription factor cDNA under the CMV promoter (Addgene) was used to obtain SP-1 overexpression.

### Transfection with small interfering RNA

Cells were transfected with 50 nM chemically synthesized siRNA targeted against human Nrf2 mRNA (sense, 5′-UGACAGAAGUUGACAAUUATT-3′; antisense, 5′-UAAUUGUCAACUUCUGUCATT-3′) or human Mxi-1 mRNA (Santa Cruz Biotechnology, Cat. No. sc-35835) or human c-Myc mRNA (Ambion, Cat. No. AM4250). As a control a scrambled siRNA (Santa Cruz Biotechnology, Cat. No. sc-37007) was used. Briefly, cells were seeded into 24-well plates one day before transfection to obtain confluence of about 50–70%. siRNA and Lipofectamine 2000 (Invitrogen) were separately diluted in Opti-MEM without serum, incubated for 5 min at room temperature, combined, and incubated for the next 20 min at room temperature. In some experiments JetPrime (Polyplus Transfection) was used instead of Lipofectamine 2000 according to the vendor's protocol. Twenty-four hours after transfection the cells were placed in a normoxic or hypoxic atmosphere for the next 24 h. For the other experiments, 24 h after transfection cells were transduced with adenoviral vectors (see below) for the next 48 h. Every 24 h the medium was replaced with fresh medium.

### Transduction with adenoviral vectors

Adenoviral vectors containing HIF-1α or HIF-2α cDNA (AdHIF-1α, AdHIF-2α) were a kind gift from Professor Seppo Yla-Herttuala and Dr. Anna Liisa-Levonen (Kuopio, Finland). The AdHIF-1α vector was generated as described previously [24]. Briefly, a construct was stabilized against prolyl hydroxylation and subsequent ubiquitin-mediated proteolytic degradation under normoxic conditions by point mutations (P402A/P563A). A control vector harboring green fluorescent protein (GFP) cDNA (AdGFP) was produced using the AdenoX system as described previously [21]. Transduction with adenoviral vectors containing cDNA for HIF-1α, HIF-2α, or GFP, as a control, was performed, and after 48 h the appropriate tests were conducted.

### Site-directed mutagenesis

Point mutations were introduced into the IL-8 promoter using the QuickChange II-E site-directed mutagenesis kit (Stratagene) according to the manufacturer's instructions. Two guanines at positions 1376 and 1377 (*Homo sapiens* IL-8 GenBank ID M28130.1), predicted as a part of the SP-1 binding site using Alibaba 2.1 computational analysis, were changed to adenine and thymine, respectively. The sequences of the sense and antisense oligonucleotides were as follow: 5′-CTCAGGTTTGCCCTGAGATGATGGGCCATCAGTTGC-3′ and 5′-GCAACTGATGGCCCATCATCTCAGGGCAAACCTGAG-3′. Introduction of mutations was verified by DNA sequencing.

### Real-time reverse transcription–PCR

Total RNA was isolated by a phenol–chloroform extraction. In brief, cells cultured in a 24-well plate were washed with phosphate-buffered saline (PBS), overlaid with 400 μl of fenozol (A&A Biotechnology), and mixed with 100 μl of chloroform. After centrifugation (30 min, 10,000 *g,* 4 °C), an upper aqueous phase was collected and subjected to ethanol precipitation. The RNA pellet was dissolved in nuclease-free water. Reverse transcription reaction was carried out on 1 μg of total RNA for 1 h at 42 °C using oligo(dT) primers and RevertAid reverse transcriptase (Fermentas), according to the vendor's instructions. Quantitative RT-PCR was performed in a StepOnePlus real-time PCR system (Applied Biosystems) in a mixture containing SYBR Green PCR Master Mix (Sigma), specific primers, and 50 ng of cDNA in a total volume of 15 μl. The EF2 housekeeping gene was used as a reference.

### Chromatin immunoprecipitation (ChIP)

Cells were cultured under standard or hypoxic conditions, and ChIP analyses were performed as previously described [25], with minor changes. Briefly, cells were cultured at 21, 5, or 0.5% O_2_ for 6 h, before they were fixed with 1% formaldehyde for 20 min. The immunoprecipitation was performed with antibodies against c-Myc (Cat. No. sc-788, Santa Cruz Biotechnology), SP-1 (Cat. No. 07–645, Millipore), or IgG (Cat. No. 309-005-003, Jackson ImmunoResearch) as a negative control. DNA was amplified by real-time PCR using the Power SYBR Green PCR Master Mix (Applied Biosystems). Primers used for PCR correspond to the putative SP-1 binding site within the IL-8 promoter (region − 121 to + 5), 5′-AGGTTTGCCCTGAGGGGATGGG-3′ and 5′-ATGGAGTGCTCCGGTGGCTTT-3′, or one of the two c-Myc binding sites within the IL-8 promoter: first (region − 802 to − 611) 5′-CTCAATGCTTGCTCCAACT-3′ and 5′-TTCTGAGTAATGTGGGGGATCT-3′, second (region − 417 to − 226) 5′-GCTGGCTTATCTTCACCATCA-3′ and 5′-GCTCCACAATTTGGTGAATTAT-3′. The mean of two experiments is shown.

### Western blotting

Cells were lysed in ice-cold PBS containing 1% Triton X-100 (Fluka) and 10 μg/ml of each of the following protease inhibitors: phenylmethylsulfonyl fluoride, leupeptin, and aprotinin (Sigma). Samples were centrifuged for 10 min, 8000 *g* at 4 °C, and clear supernatants were collected. 30–50 μg of protein was loaded onto a 10% SDS–PAGE gel followed by 2.5 h electrotransfer to nitrocellulose membrane Protran (PerkinElmer Life Sciences). After blocking in 5% nonfat milk for 1 h at room temperature, membranes were probed with polyclonal antibodies against HIF-1α (Cat. No. sc-10790), HIF-2α (Cat. No. sc-28706), Nrf2 (Cat. No. sc-13032), Bach1 (Cat. No. sc-14699), c-Myc (Cat. No. sc-788) (Santa Cruz Biotechnology); HO-1 (Stressgen, Cat. No. SPA-894); or α-tubulin (Calbiochem, Cat. No. CP06), as a loading control, overnight at 4 °C, followed by HRP-linked secondary antibodies (Cell Signaling Technology) for 45 min at room temperature. All antibodies were diluted in 0.05% Tween containing PBS with 5% nonfat dry milk. Visualization was performed using SuperSignal West Pico chemiluminescence substrate (Pierce Biotechnology) according to the manufacturer's instructions.

### Measurement of protein synthesis by ELISA

IL-8 and VEGF concentrations in the cell culture medium were determined using ELISA according to the vendor's protocols (R&D Systems).

### Immunofluorescence for detection of HIF α isoforms

Cells were grown on chamber slides under standard or hypoxic conditions for 24 h and then they were fixed in 4% formaldehyde for 30 min at room temperature. After being washed with PBS containing ions, the cells were permeabilized using 0.2% Triton X-100 for 20 min at room temperature and then blocked with 10% goat serum for 1 h. Slides were probed with polyclonal antibodies against HIF-1α or HIF-2α (Santa Cruz Biotechnology) overnight at 4 °C. After a wash with PBS, secondary polyclonal FITC (green) antibody (Cappel) or Alexa Fluor 546 (red) antibody (Invitrogen) was added together with 1 μg/ml Hoechst dye (for nuclear staining-blue; Sigma) for 1 h at room temperature. All antibodies were diluted in 5% goat serum in PBS. Cells were washed with PBS and observed under fluorescence microscope (Nikon).

### Statistical analysis

All experiments were performed in duplicate and most were repeated three times. All data are presented as means ± standard deviation (SD) and were analyzed by analysis of variance followed by a Bonferroni post hoc test for multiple comparisons or by a Student *t* test for two-group comparisons. Differences were accepted as statistically significant at *p* < 0.05.

## Results

### Opposite effects of HIF-1α and HIF-2α on IL-8 expression

**Fig. 1:**
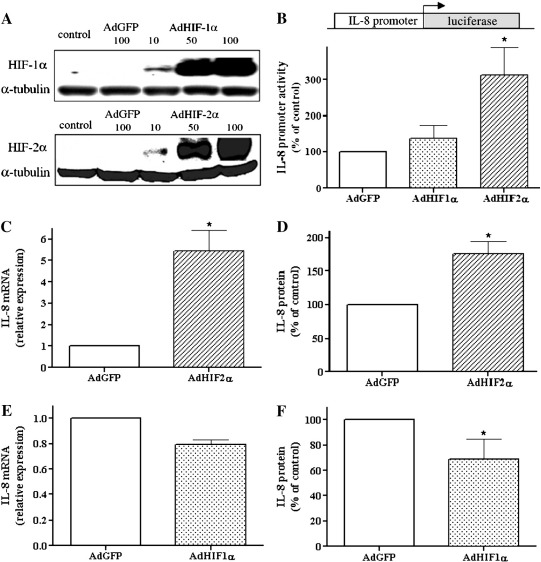
Opposite effects of HIF-1α and HIF-2α on IL-8 expression. (A) Cells were transduced for 48 h with 10, 50, and 100 MOI of AdHIF-1α and AdHIF-2α as well as 100 MOI of AdGFP as a control. Nontransduced cells were used as an additional control. Overexpression of HIF-1α and HIF-2α was confirmed by Western blotting; representative immunoblots are shown. (B) Cells were cotransfected with plasmid containing the full-length promoter of the IL-8 gene driving luciferase expression (500 ng) and plasmid containing the LacZ gene (100 ng) as an internal control and after 24 h were transduced with AdHIF-1α, AdHIF-2α, and AdGFP for the next 48 h. Next, the activity of luciferase was measured. IL-8 promoter activity was potently induced in response to HIF-2α overexpression. (C, E) Real-time PCR was performed to examine the IL-8 mRNA level after transduction with HIF-1α or HIF-2α adenoviral vectors. (D, F) ELISA was performed to assess the IL-8 protein level after 48 h transduction with adenoviral vectors. In contrast to AdHIF-1α, transduction with AdHIF-2α increased IL-8 expression at the level of both mRNA and protein. Each bar represents the mean ± SD of two to six independent experiments performed in duplicate. *p < 0.05, comparing AdGFP vs AdHIF-1α or AdHIF-2α.

To obtain HIF-α isoform overexpression, HMEC-1 cells were transduced with adenoviral vectors carrying HIF-1α or HIF-2α cDNA. Increased levels of HIF proteins at various multiplicities of infection (MOI) of the adenoviral vectors (10, 50, 100) were confirmed by Western blotting (Fig. 1A). As controls, AdGFP-treated and nontransduced cells were used. As expected, AdHIF-1α and AdHIF-2α significantly increased in a concentration-dependent manner the protein levels of HIF-1α and HIF-2α, respectively, compared to AdGFP-transduced and nontransduced cells (Fig. 1A). The high efficiency of transduction was confirmed by detection of GFP expression (not shown).

We have recently shown that expression of IL-8 is downregulated by hypoxia or HIF-1α in an Nrf2-dependent manner [21]. Because HIF isoforms may exert different effects, we aimed to examine the influence of HIF-2α on IL-8 expression. HMEC-1 cells were transiently transfected with plasmid containing the full-length promoter of the IL-8 gene driving luciferase expression and subsequently transduced with AdHIF-1α, AdHIF-2α, or AdGFP as a control. Interestingly, the activity of luciferase was strongly enhanced after HIF-2α, but just slightly and insignificantly after HIF-1α (Fig. 1B). Accordingly, HIF-2α increased IL-8 expression at the level of mRNA and protein, as shown by real-time PCR and ELISA, respectively (Figs. 1C and D). In contrast, AdHIF-1α diminished IL-8 mRNA and protein (Figs. 1E and F). Of note, neither HIF-1α nor HIF-2α overexpression caused changes in the expression of other proinflammatory cytokines, IL-6 and IL-1β, as tested by ELISA (not shown).

**Fig. 2:**
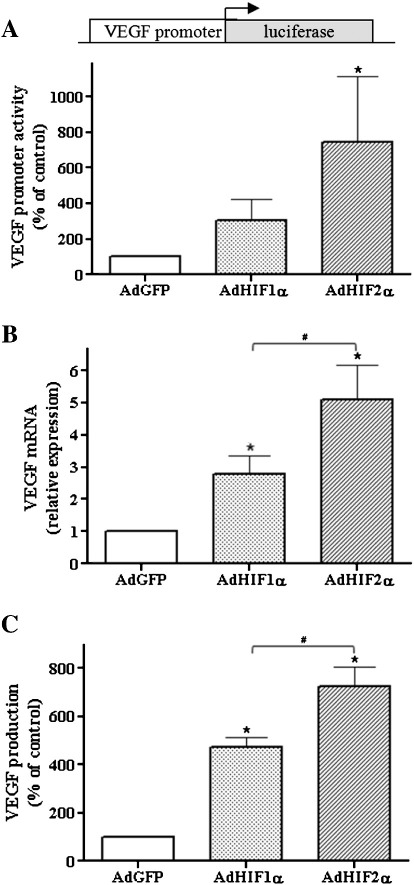
Increased expression of VEGF after HIF-1α and HIF-2α. (A) Cells were cotransfected with plasmid containing the full-length promoter of the VEGF gene driving luciferase expression (500 ng) and plasmid containing the LacZ gene (100 ng) as an internal control and after 24 h transduced with AdHIF-1α, AdHIF-2α, or AdGFP for the next 48 h. As shown by measurement of luciferase activity, both HIF-1α and HIF-2α activated VEGF transcription. (B) Real-time PCR and (C) ELISA were performed to examine the mRNA and protein level of VEGF, respectively, after 48 h of transduction with adenoviral vectors. Both HIF isoforms enhanced VEGF production, but the effect of HIF-2α was more potent. Each bar represents the mean ± SD of three to six independent experiments performed in duplicate. *p < 0.05, comparing AdGFP vs AdHIF-1α or AdHIF-2α, #*p* < 0.05, comparing AdHIF-1α vs AdHIF-2α.

To confirm the efficiency of adenoviral vector transduction we checked the expression of VEGF, a known common target gene of HIF isoforms. Studies using the construct containing the full-length promoter of the VEGF gene driving luciferase expression showed that both HIF-1α and HIF-2α activate VEGF transcription (Fig. 2A). The levels of mRNA (Fig. 2B) and protein (Fig. 2C) were also increased. Notably, HIF-2α induced VEGF expression more potently than HIF-1α at both mRNA and protein levels (Figs. 2B and C).

### HIF-2α increases IL-8 expression independent of Nrf2

**Fig. 3:**
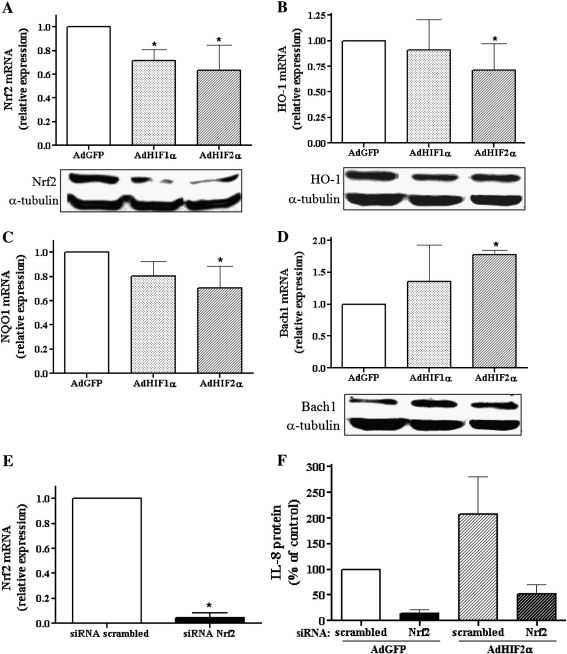
HIF-2α increases IL-8 expression independent of Nrf2. (A–D) Cells were transduced for 48 h with AdHIF-1α, AdHIF-2α, or AdGFP as a control. Real-time PCR was performed to examine the mRNA levels of Nrf2, HO-1, NQO1, and Bach1. Western blotting was done using antibodies against Nrf2, HO-1, and Bach1; representative immunoblots are shown. Note a decrease in Nrf2 and its target genes’ expression and a concomitant increase in the expression of Bach1, the Nrf2 repressor. (E) Real-time PCR after 48 h transfection with siRNA targeted against Nrf2 mRNA or scrambled siRNA. Nrf2 siRNA abolished the Nrf2 mRNA expression. (F) ELISA was performed to assess the protein level of IL-8 after 24 h transfection with siRNA against Nrf2 mRNA or scrambled siRNA followed by 48 h transduction with AdHIF-2α or AdGFP. Silencing of Nrf2 did not influence HIF-2α-induced IL-8 production compared to cells transduced with the AdGFP vector, in which Nrf2 was also silenced. Each bar represents the mean ± SD of two to six independent experiments performed in duplicate. *p < 0.05, comparing AdGFP vs AdHIF-1α or AdHIF-2α.

HIF-1α decreases the IL-8 level by inhibiting the Nrf2 transcription factor [21], a known regulator of antioxidant genes including heme oxygenase-1 (HO-1) (for review see [26]). Therefore, we examined if the upregulation of IL-8 by HIF-2α can be mediated by an increase in Nrf2. The obtained results showed that, at both the mRNA and the protein level, Nrf2 is downregulated by HIF-1α and HIF-2α (Fig. 3A). In accordance, the expression of HO-1 and NAD(P)H:quinone oxidoreductase (NQO1), the other known target gene of Nrf2, was also reduced (Figs. 3B and C, respectively). Furthermore, after transduction with either of the HIF α subunits we observed a concomitant increase in the expression of Bach1, the Nrf2 repressor (Fig. 3D). Therefore, similar inhibitory effects of HIF-1α and HIF-2α on Nrf2 suggest that the HIF-2α-induced enhancement of IL-8 level is not dependent on Nrf2.

To further confirm this supposition, cells were transfected with 50 nM chemically synthesized siRNA targeted against human Nrf2 mRNA (Fig. 3E) and subsequently transduced with AdHIF-2α or AdGFP as a control. Inhibition of Nrf2 significantly diminished the expression of IL-8, in both cells transduced with either AdGFP or AdHIF-2α (Fig. 3F). However, it did not attenuate the upregulation rate of IL-8 in response to HIF-2α (2.07- and 5.21-fold increase in scrambled siRNA and siRNA against Nrf2-treated cells, respectively). This confirms the important role of Nrf2 in regulation of IL-8 expression but not as an effector in the HIF-2-dependent pathway.

### HIF-2α overexpression increases SP-1 activity

**Fig. 4:**
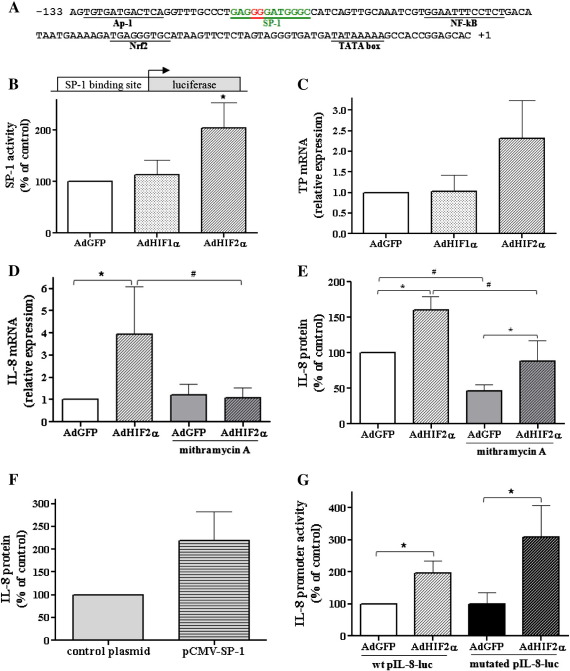
HIF-2α overexpression increases SP-1 activity. (A) SP-1 transcription factor binding site found in the promoter of IL-8 using the Alibaba 2.1 program. (B) Cells were cotransfected with the pSP-1-luc vector containing the SP-1 binding site driving luciferase expression (500 ng) and a plasmid containing the LacZ gene (100 ng) as an internal control and after 24 h transduced with AdHIF-1α, AdHIF-2α, or AdGFP for the next 48 h. As shown by measurement of luciferase activity, HIF-2α augmented SP-1 activity. (C) Real-time PCR was performed to examine the mRNA level of a TP gene containing binding sites for SP-1 after 48 h transduction with AdHIF-1α, AdHIF-2α, or AdGFP. TP mRNA was upregulated only by HIF-2α (a similar tendency of increase in TP was noted in three independent experiments). (D) Real-time PCR and (E) ELISA were performed to examine the mRNA and protein level of IL-8, respectively, after 48 h transduction with AdHIF-2α or AdGFP together with stimulation with 10 μM SP-1 inhibitor, mithramycin A. Note that the effect of HIF-2α on IL-8 was partially reversed by mithramycin A, particularly at the mRNA level. (F) Cells were transfected for 48 h with the pCMV-SP-1 vector (500 ng) or control Bluescript plasmid (100 ng). As shown by ELISA, SP-1 overexpression tended to increase IL-8 production. (G) SP-1 transcription factor binding site found in the promoter of IL-8 was mutated at guanine residues (in red in (A)). Such mutation did not change HIF-2α-induced IL-8 promoter activity. Each bar represents the mean ± SD of three to six independent experiments performed in duplicate. *p < 0.05, comparing AdGFP vs AdHIF-1α or AdHIF-2α; #*p* < 0.05, comparing AdGFP control vs AdGFP stimulated or AdHIF-2α control vs AdHIF-2α stimulated.

An analysis of the IL-8 promoter revealed the presence of consensus sequences of the Nrf2, NF-κB, AP-1, and SP-1 transcription factors (Fig. 4A). To investigate their involvement in regulation of IL-8 expression we used the pNFκB-SEAP and pAP-1-SEAP vectors, containing the NF-κB and AP-1 binding regions connected to the secreted alkaline phosphatase reporter gene, as well as the pSP-1-luc and pARE-luc vectors containing the SP-1 binding site or ARE (Nrf2 binding site), respectively, driving luciferase expression. After transfection, the cells were transduced with adenoviral vectors for 48 h and subsequently reporter gene expression was examined. In case of pNFκB-SEAP and pAP-1-SEAP we did not observe any significant differences in SEAP activity between cells transduced with AdGFP or AdHIF-1α or AdHIF-2α (not shown). Importantly, luciferase activity after transfection with the pSP-1-luc vector was significantly increased in response to HIF-2α overexpression in comparison to GFP or HIF-1α (Fig. 4B). Furthermore, thymidine phosphorylase (TP), an example of an SP-1 target gene, containing binding sites for SP-1 in its promoter region, tended to be upregulated only by HIF-2α, as shown by real-time PCR (Fig. 4C). For inhibition of SP-1 activity 10 μM mithramycin A was applied 24 h before the end of the experiment. Dimethyl sulfoxide at an appropriate concentration was included in the control. Importantly, the effect of HIF-2α on IL-8, as well as TP expression, was reversed by mithramycin A (Figs. 4D and 3E; not shown for TP).

To further examine the role of SP-1 in HIF-2α-mediated upregulation of IL-8 we overexpressed SP-1 using the pCMV-SP-1 plasmid. As expected, SP-1 overexpression tended to increase IL-8 production (Fig. 4F). Then we performed site-directed mutagenesis and mutated two guanine residues in the predicted SP-1 binding site in the promoter region of IL-8. However, after transfection of HMEC-1 cells with the mutated form of the pIL-8-luc vector, we did not observe a decrease in the level of IL-8 promoter activity after subsequent transduction with AdHIF-2α in comparison to the wild-type vector (Fig. 4G). This suggests that the effect of SP-1 on IL-8 expression may be rather indirect.

### Severe and mild hypoxia evoke similar effects on IL-8 expression

The level of oxygen in different tissues can vary and the influence of moderate and strong hypoxia on gene expression can be different. Indeed, HIF-2 was suggested to mediate hypoxic responses under moderate hypoxia (5% O_2_), and HIF-1 may be stabilized at lower oxygen tension (reviewed in [27]). Hence, one may expect that the opposite effects of two HIF isoforms may be detected under different hypoxia levels.

**Fig. 5:**
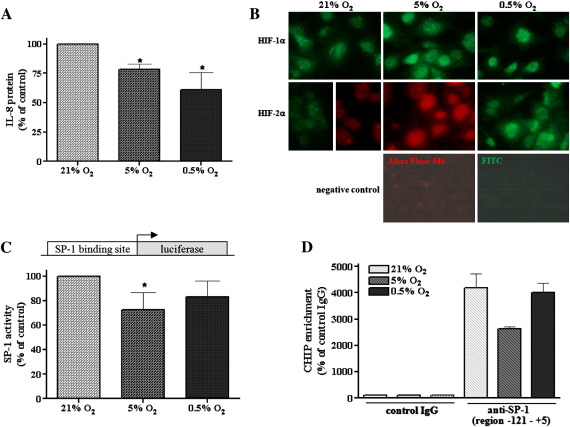
Effect of severe (0.5% O_2_) and mild (5% O_2_) hypoxia on IL-8 expression and SP-1 activity. (A) ELISA was done to assess the IL-8 protein level after 24 h of normoxia (21% O_2_) and mild (5% O_2_) and severe (0.5% O_2_) hypoxia. Note the decrease in IL-8 production under both conditions. (B) As shown by immunocytochemistry, both HIF subunits were activated in HMEC-1 cells under 0.5 and 5% O_2_; representative staining is shown (original magnification 100×). (C) Cells were cotransfected with pSP-1-luc vector containing the SP-1 binding site driving luciferase expression (500 ng) and a plasmid containing the LacZ gene (100 ng) as an internal control and after 24 h incubated under 21, 5, or 0.5% O_2_ for the next 24 h. Then the luciferase activity was measured. (D) ChIP assay was done to check the SP-1 binding to the IL-8 promoter under severe and mild hypoxia (6 h). Antibodies against IgG and SP-1 (anti-SP-1) were used. The SP-1 binding site within the IL-8 promoter was amplified using real-time reverse transcription–PCR as described under Materials and methods. Both SP-1 activity (C) and SP-1 binding to the IL-8 promoter (D) were slightly affected under both hypoxic conditions, but particularly under 5% O_2_. Each bar represents the mean ± SD of two to five independent experiments. *p < 0.05, comparing 21% vs 0.5 or 5% O_2_.

In our hands, however, the production of IL-8 was decreased to similar extents by both 0.5 and 5% oxygen (Fig. 5A). Furthermore both, HIF-1α and HIF-2α were activated in HMEC-1 cells under both severe (0.5% O_2_) and mild (5% O_2_) hypoxia (Fig. 5B). Accordingly, we showed that SP-1 activity detected in the reporter gene assay (Fig. 5C), as well as SP-1 binding to the IL-8 promoter, revealed by ChIP analysis (Fig. 5D), was slightly altered under both hypoxic conditions, but particularly under 5% O_2_.

### c-Myc and Mxi-1 are involved in different regulation of IL-8 expression by HIF isoforms

Recent studies by Yoo and coworkers revealed that IL-8 is upregulated by the silencing of Mxi-1, a c-Myc antagonist [20]. Moreover, HIF-2α was shown to increase, whereas HIF-1α decreased, c-Myc activity by inducing Mxi-1 [18] or by alteration of the interaction with SP-1, a coactivator of c-Myc [16]. Finally, Mxi-1 is induced by hypoxia, in a HIF-1α-dependent manner, with concomitant downregulation of c-Myc target genes [19,20]. Thus, we hypothesized that the observed effects of HIF isoforms on IL-8 expression may be related to Mxi-1/c-Myc proteins.

**Fig. 6:**
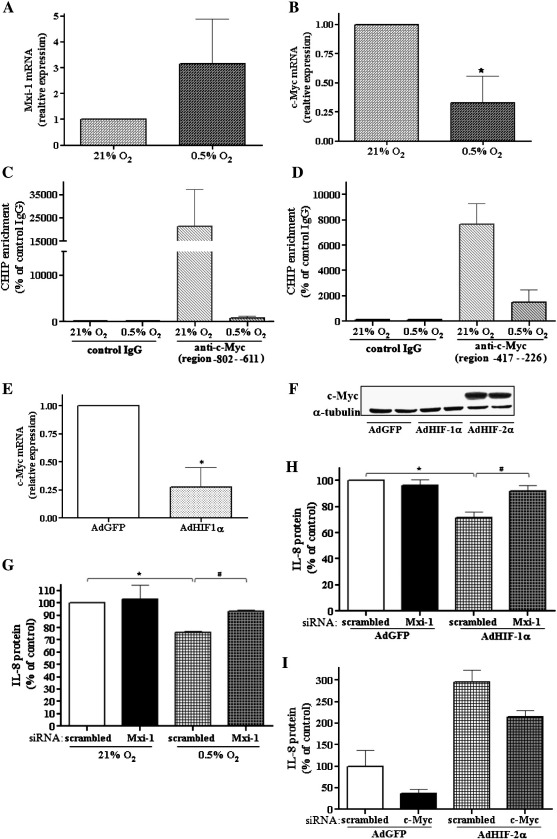
c-Myc and Mxi-1 are involved in different regulation of IL-8 expression by HIF isoforms. Real-time PCR was performed to examine (A) Mxi-1 and (B) c-Myc mRNA after 24 h incubation of cells under severe (0.5% O_2_) hypoxia. Note the tendency toward increase in Mxi-1 and decrease in c-Myc expression under hypoxia. (C, D) ChIP assay was performed to check c-Myc binding to the IL-8 promoter ((C) region − 802 to − 611 and (D) region − 417 to − 226) under hypoxic conditions (6 h). Antibodies against IgG and c-Myc (anti-c-Myc) were used. Binding sites of c-Myc within the IL-8 promoter were amplified using real-time PCR as described under Materials and methods. Note a strong decrease in c-Myc binding under hypoxic conditions. (E) Real-time PCR was performed to examine c-Myc mRNA after 48 h transduction with AdHIF-1α. AdHIF-1α diminished c-Myc mRNA. (F) Cells were transduced for 48 h with AdHIF-1α, AdHIF-2α, or AdGFP as a control. As shown by Western blotting, in contrast to HIF-1α, overexpression of HIF-2α resulted in increased expression of c-Myc protein; the representative immunoblot is shown. (G) ELISA was performed to assess the IL-8 protein level after 24 h transfection with siRNA against Mxi-1 mRNA or scrambled siRNA as a control and incubation under normoxic or hypoxic conditions for the next 24 h. (H) ELISA was done to assess the IL-8 protein level after 24 h transfection with siRNA against Mxi-1 mRNA or scrambled siRNA as a control and 48 h transduction with AdHIF-1α or AdGFP. Silencing of Mxi-1 by siRNA reversed both the AdHIF-1α- and the hypoxia-dependent diminishment of IL-8. (I) ELISA was done to assess IL-8 protein level after 24 h transfection with siRNA against c-Myc mRNA or scrambled siRNA as a control and 48 h transduction with AdHIF-2α or AdGFP. c-Myc siRNA partially reversed HIF-2α-induced IL-8 expression. Each bar represents the mean ± SD of two to four independent experiments performed in duplicate or (I) a representative experiment is shown. *p < 0.05, comparing normoxia vs hypoxia or AdGFP vs AdHIF-1α or AdHIF-2α; #*p* < 0.05, comparing scrambled siRNA vs siRNA against Mxi-1.

We observed a tendency of augmentation of Mxi-1 in severe hypoxia (0.5% O_2_) and a significant decrease in c-Myc expression (Figs. 6A and B, respectively). In accordance with the latter, using a ChIP assay, we demonstrated that c-Myc binding to both binding sites within the IL-8 promoter (region − 802 to − 611 and − 417 to − 226) is markedly decreased under hypoxic conditions (Figs. 6C and D).

Moreover, transduction of cells with AdHIF-1α diminished c-Myc (Fig. 6E). Transduction of cells with AdHIF-2α did not influence the level of c-Myc mRNA (not shown), but, in contrast to HIF-1α, overexpression of the HIF-2α isoform resulted in increased expression of the c-Myc protein (Fig. 6F). To further confirm the involvement of Mxi-1 in the effect of HIF-1α on the decrease of IL-8 expression we used siRNA against Mxi-1 and incubated cells under severe hypoxia (0.5% O_2_) or transduced them with AdHIF-1α. In both cases, siRNA transfection reversed the downregulation of IL-8 (Figs. 6G and H). On the other hand, treatment of cells with siRNA against c-Myc partially reversed the induction of IL-8 expression after AdHIF-2α (Fig. 6I).

## Discussion

The salient finding of this study is the demonstration of the different roles of HIF isoforms in regulation of IL-8 expression in endothelial cells. In contrast to HIF-1α, overexpression of HIF-2α results in a significantly increased level of IL-8. We suggest the involvement of SP-1 and c-Myc in the HIF-2α-dependent IL-8 upregulation, whereas inhibition of both SP-1 and c-Myc, as well as upregulation of Mxi-1, a c-Myc antagonist, mediates the action of hypoxia/HIF-1α. Moreover, despite stabilization of both isoforms in hypoxia the effect of HIF-1α is predominant and downregulation of IL-8 expression in hypoxia is caused by attenuation of Nrf2 [21] and c-Myc.

HIF-1α and HIF-2α are the most extensively studied and understood of the three HIF α isoforms. HIF-1α has been recognized to control more than 100 target genes, and more than 2% of all human genes are suggested to be either directly or indirectly regulated by this factor in endothelial cells [28]. Recent reports have also revealed a number of selective HIF-2α-responsive genes [13,29]. Although HIF-1α and HIF-2α share significant sequence homology, they have unique tissue distributions, embryonic deletion phenotypes, and effects on blood vessel formation during tumorigenesis [10–15,30]. Accordingly, differences exist between the transactivation domains of HIF isoforms [5], implying HIF-1α and HIF-2α may require different transcriptional cofactors to regulate distinct target genes. Interestingly, in addition to coactivators shared by both HIF isoforms, specific ones have been found, which take part exclusively in the formation of a transcriptional complex regulating target genes of HIF-2α.

We revealed IL-8 as a new candidate for differential regulation by HIF-1α and HIF-2α. Recently we have shown that expression of IL-8 is reduced after overexpression of the stable form of HIF-1α or under hypoxic conditions [21]. In this study we report the opposite action of HIF-2α, which strongly upregulated the expression of IL-8 at the levels of promoter activity, mRNA, and protein. HIF-1α decreased the IL-8 level by inhibiting Nrf2 expression and activity, with the concomitant induction of Bach1, a repressor of Nrf2 transcriptional activity. Similarly, the expression of HO-1 was affected by HIF-1α via Nrf2 inhibition [21]. However, the effect of HIF-1 on IL-8 expression is not mediated by HO-1. Our data indicate that an increase in IL-8 after AdHIF-2α was not associated with Nrf2, because we observed a similar downregulation of Nrf2 and upregulation of Bach1 after overexpression of both HIF-1α [21] and HIF-2α (this study). Moreover, searching for other mediators of HIF-2α, we excluded AP-1 and NF-κB, transcription factors known to regulate IL-8 expression. Instead, the activity of SP-1, whose binding site is present in the promoter of IL-8, was augmented after AdHIF-2α transduction. Furthermore, the effect of AdHIF-2α on IL-8 upregulation was reversed by the SP-1 inhibitor mithramycin A, thereby suggesting the SP-1-dependent induction of IL-8 by HIF-2α. Interestingly, SP-1 direct binding to the IL-8 promoter was not fundamental for HIF-2α action, as revealed by site-directed mutagenesis and experiments with a mutated SP-1 binding site. Thus, in addition to binding of SP-1 to the IL-8 promoter under basal conditions other factors are probably involved in the upregulation of IL-8 in response to HIF-2α overexpression. Indeed, our data suggest that diminishment of IL-8 by hypoxia or HIF-1 and acceleration of IL-8 expression by HIF-2 might be dependent on the opposite effects on c-Myc activity.

The c-Myc transcription factor regulates the expression of genes involved in cell proliferation, apoptosis, glucose, and energy metabolism and is overexpressed in many tumors [31]. It heterodimerizes with the Max protein and binds to consensus E-box elements in the target genes [32]. Transcriptional repressors belonging to the Mad family (Mad1, Mad2/Mxi1, Mad3, Mad4) are known to be regulated inversely compared to c-Myc protein [4,33].

It has been shown that HIF-2α promotes cell-cycle progression in hypoxic renal cell carcinoma (RCC) and multiple other cell lines. Importantly, this correlated with enhanced c-Myc promoter binding, transcriptional effects on both activated and repressed target genes, and interactions with SP-1, Miz1, and Max [16]. Thus, HIF-2α has been shown to enhance c-Myc activity. HIF-1α, on the other hand, acted in the opposite way and inhibited c-Myc function, causing cell cycle arrest at the G1/S phase. In their study on RCC, Gordan and coworkers [16] proposed that HIF-1α specifically disrupts c-Myc:Max and c-Myc:SP-1 complexes, allowing more Mad:Max interaction and DNA binding. On the other hand, they hypothesized that HIF-2α stabilizes c-Myc:Max complexes, in turn promoting c-Myc DNA binding at E boxes [16]. In another study by Lofsted and coworkers [18] it was demonstrated that hypoxia upregulates Mxi-1 mRNA and protein in neuroblastoma and breast cancer cells, and Mxi-1 was confirmed as a direct HIF-1α target gene. In a murine hepatoma cell line Mxi-1 induction by hypoxia was also found to be HIF-1-dependent and caused concomitant downregulation of the c-Myc target genes [19]. Here we also show that hypoxia augments Mxi-1 and simultaneously diminishes c-Myc expression in HMEC-1 cells. In accordance, transduction of cells with AdHIF-1α decreased c-Myc levels, suggesting that the effect of hypoxia is primarily mediated by HIF-1α.

Interestingly, recent studies by Yoo and coworkers demonstrated that IL-8 is upregulated in Mxi-1 knockdown cells [20]. Moreover, by using computational analysis we found two c-Myc:Max binding sites in the promoter region of IL-8, which suggests that IL-8 might be directly regulated by c-Myc. Indeed, using ChIP analysis, we reported that c-Myc binds to the promoter region of IL-8 under normoxic conditions and this interaction is strikingly inhibited in response to hypoxia. Thus, we postulate that the observed effects of HIF isoforms on IL-8 expression are related to Mxi-1/c-Myc interactions and/or their interaction with SP-1. We report also that Mxi-1 is involved in the effect of HIF-1α because the silencing of Mxi-1 reversed IL-8 downregulation after hypoxia or AdHIF-1α transduction. On the other hand, IL-8 upregulation in response to HIF-2α is at least partially mediated by c-Myc because siRNA against the latter reversed the induction of IL-8 expression.

**Fig. 7:**
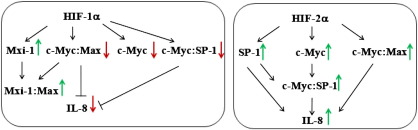
Proposed mechanism of regulation of IL-8 expression by interaction of c-Myc/Max, SP-1, and HIF-1α/HIF-2α.

Taken together, our data show that Mxi-1 is involved in downregulation of IL-8 expression after HIF-1α overexpression as well as by hypoxia in HMEC-1 cells. The possible mechanism may be related to an increase in Mxi-1 level, a decrease in c-Myc, or disruption of c-Myc:SP-1 and/or c-Myc:Max complex formation. On the other side, overexpression of HIF-2α results in upregulation of IL-8 expression through induction of SP-1 activity, increase in the c-Myc level, or stimulation of c-Myc:SP-1 and/or c-Myc:Max complex formation (Fig. 7). Given the opposing roles of HIF isoforms on c-Myc transcriptional activity and IL-8 level, the functional outcome of their hypoxic regulation may depend on the relative expression levels of HIF-1α and HIF-2α in a given cell type, especially if they may also both be activated under severe (0.5% O_2_) and more physiological (5.0% O_2_) hypoxia as well. To modulate vascular growth and cancer cell metabolism and survival, and in turn to establish new therapies, it is reasonable to further examine the reciprocal ratio of HIF isoforms in a particular cell type or tumor in which the coactivation of both isoforms is usual.

The data presented here suggest some relevance to tumor growth and antiangiogenic therapies. The strategies based on HIF-1 inhibition may lead to downregulation of some genes (such as VEGF) but concomitantly IL-8 may be increased. These results are in accordance with the experiments of Mizukami et al. [34], who observed no difference between vascularization of tumors formed after injection of either wild-type or HIF-1α knockdown colon cancer cells into nude mice. In fact, in such cells, despite the inhibition of VEGF, the IL-8 synthesis was upregulated [34].

However, in some tumors, e.g., human glioblastoma, expression of HIF-2α is particularly high and it plays a more important role than HIF-1α [35]. Therefore, it would be reasonable to investigate if such tumors are characterized by increased IL-8 production. Then the strategy of inhibition of HIF-2α might be considered as additional anticancer therapy. Interestingly, mithramycin A, which was able to reverse the effect of HIF-2α on IL-8 expression, is used as an anticancer drug in clinics. Although the mechanism of its action is not fully understood, it is known, that mithramycin A via triggering activity of p53 protein and inhibition of SP-1, decreases angiogenesis in tumors [36,37].

An aberrant expression of angiogenic mediators contributes to the development and progression of numerous diseases. This study, in revealing the opposite roles played by HIF-1α and HIF-2α in the regulation of IL-8 expression in endothelial cells, points to the potential limitations of antiangiogenic strategies targeted at a single transcription factor.
